# Olfactory Impairment in Persons Living with Dementia

**DOI:** 10.1002/alz70857_101396

**Published:** 2025-12-25

**Authors:** Alex Bahar‐Fuchs

**Affiliations:** ^1^ Deakin University, Melbourne, VIC, Australia

## Abstract

A strong link between a decline in olfactory functions and neurodegenerative diseases such as Alzheimer's and Lewy body disease has been well established for several decades. By the time dementia is diagnosed, olfactory impairment is present in as many as 90% of patients. An impaired sense of smell in people with dementia is associated with additional decline in functional independence and places patients at risk due to inability to correctly identify smells, particularly those signalling risk (e.g., smoke, gas). There is therefore great importance in the identification and management of impaired olfaction, however this is rarely done in routine assessments of people with or at risk of dementia. Unfortunately, despite a strong interest in recent years in the links between sensory decline and dementia, olfaction remains a relatively neglected area of research and clinical practice relative to the more recently established links between visual and auditory decline and with dementia. In this talk, I will first briefly discuss the role played by the sense of smell in a range of everyday activities and behaviours and outline the shared neuroanatomical pathways between olfactory processes and cognition. I will present consistent evidence confirming that olfactory impairment is a prodromal symptom in the pre‐dementia phase of many neurodegenerative conditions and a strong predictor of conversion from cognitive health to mild cognitive impairment (MCI) and from MCI to dementia. I will then describe the key tools for the assessment of olfaction which may be used in the clinic. Finally, I will describe the burgeoning literature on the training of the sense of smell and present compelling evidence that training of the sense of smell is associated with improvements in cognition, as demonstrated in the recently completed Mind Your Nose trial.

